# Detection of Craving for Gaming in Adolescents with Internet Gaming Disorder Using Multimodal Biosignals

**DOI:** 10.3390/s18010102

**Published:** 2018-01-01

**Authors:** Hodam Kim, Jihyeon Ha, Won-Du Chang, Wanjoo Park, Laehyun Kim, Chang-Hwan Im

**Affiliations:** 1 Department of Biomedical Engineering, Hanyang University, Seoul 04673, Korea; rhg910907@hanyang.ac.kr (H.K.); hj910410@kist.re.kr (J.H.); 2 Center for Bionics, Korea Institute of Science and Technology, Seoul 02792, Korea; wanjoo.park@gmail.com (W.P.); laehyunk@kist.re.kr (L.K.); 3 School of Electronic and Biomedical Engineering, Tongmyong University, Busan 48520, Korea; 12cross@gmail.com

**Keywords:** internet gaming disorder, internet game addiction, biosignal analysis, craving, machine learning

## Abstract

The increase in the number of adolescents with internet gaming disorder (IGD), a type of behavioral addiction is becoming an issue of public concern. Teaching adolescents to suppress their craving for gaming in daily life situations is one of the core strategies for treating IGD. Recent studies have demonstrated that computer-aided treatment methods, such as neurofeedback therapy, are effective in relieving the symptoms of a variety of addictions. When a computer-aided treatment strategy is applied to the treatment of IGD, detecting whether an individual is currently experiencing a craving for gaming is important. We aroused a craving for gaming in 57 adolescents with mild to severe IGD using numerous short video clips showing gameplay videos of three addictive games. At the same time, a variety of biosignals were recorded including photoplethysmogram, galvanic skin response, and electrooculogram measurements. After observing the changes in these biosignals during the craving state, we classified each individual participant’s craving/non-craving states using a support vector machine. When video clips edited to arouse a craving for gaming were played, significant decreases in the standard deviation of the heart rate, the number of eye blinks, and saccadic eye movements were observed, along with a significant increase in the mean respiratory rate. Based on these results, we were able to classify whether an individual participant felt a craving for gaming with an average accuracy of 87.04%. This is the first study that has attempted to detect a craving for gaming in an individual with IGD using multimodal biosignal measurements. Moreover, this is the first that showed that an electrooculogram could provide useful biosignal markers for detecting a craving for gaming.

## 1. Introduction

Internet gaming disorder (IGD, also referred to as internet game addiction) was included in the Diagnostic and Statistical Manual of Mental Disorder-V (DSM-V) in 2013 as a behavioral addiction [1]. In particular, IGD in adolescents is becoming an issue of public concern. Common key elements of substance use disorders (SUDs) and behavioral addictions are craving, impaired control and continued behavioral engagement [2]. Among these, craving, which is defined as “the accompanied emotional state or an extreme desire that is produced by conditioned stimuli that are associated with the reward effects of substances or behavior” [3], has been shown to play a core role in the continuation of behavioral addictions [4,5].

Numerous studies have reported the brain reactivity and autonomic nervous system responses evoked by addiction-related cues or stimuli. The brain regions that were commonly reported in different addiction studies include the nucleus accumbens, amygdala, striatum, anterior cingulate cortex, orbitofrontal cortex, and dorsolateral prefrontal cortex [6,7]. A person’s heart rate and skin conductance also change when they feel a strong craving for some object [8,9,10,11]. Because internet game addiction is a relatively new type of addiction, there have only been a few studies reporting the changes in the biosignals of individuals with IGD. Lu et al. investigated the changes in four biosignals, including the blood volume pulse, skin conductance, peripheral temperature, and respiratory response, while individuals at a high risk of developing IGD were using the internet. Their results consistently showed increases in blood volume, body temperature, and respiratory rate, as well as a decrease in skin conductance, suggesting that the sympathetic nervous system is excessively activated in individuals with a high risk of developing IGD [12]. More recently, Hsieh and Hsiao observed similar trends in internet abusers when emotional film clips were presented [13].

On the other hand, recent studies have demonstrated that computer-aided treatment methods, such neurofeedback therapy, could be effective in treating various types of addiction disorders [14,15,16]. To implement more effective computer-aided methods for the treatment of addiction, estimating whether an individual is currently craving something would be of great help. For example, this information could be used to monitor whether a current treatment program is effective in reducing the craving for the target object or to provide specific feedback for neurofeedback-based treatments. To the best of our knowledge, however, no studies have attempted to detect a craving for gaming in individuals with IGD using biosignal changes.

In this study, two kinds of videos were presented to the study participants while measuring their photoplethysmogram (PPG), galvanic skin response (GSR), and electrooculogram (EOG) signals. One video clip showed scenes from addictive online games and the other showed natural scenery. We confirmed how much craving the participant felt for gaming using a self-report craving score, and then observed the subsequent changes in biosignals recorded during the gameplay video screening. Finally, we applied a support vector machine (SVM) to classify the craving states of each individual using features extracted from the multimodal biosignals. Among the various biosignals adopted in this study, EOG has never been considered as a potential marker of the craving state in individuals with IGD. We selected EOG signals as they have the potential to reflect biased attentional orientation to targets of addiction.

## 2. Materials and Methods

### 2.1. Participants

A total of 62 male participants (age: 19.31 ± 2.51 years) participated in our experiment. The severity of their game addiction (Young scale) was individually evaluated using Young’s Internet Addiction Test [17,18]. An expert psychiatrist who had been researching this area for more than two years screened the participants, and applied the Young’s test. Before the experiment, all the experimental procedures were explained to the participants or their legal guardians, and informed consent was obtained from all of them. They received a monetary reimbursement after participating in the experiment. Five participants were excluded in the further analysis procedure because of a failure to record clean artefact-free biosignals (57 participants, age: 19.19 ± 2.49 years, Young scale: 48.23 ± 18.65). Supplementary Tables S1 and S2 show the individual Young scales of 57 participants. This experimental study was approved and reviewed by the Institutional Review Board (IRB) of the Korea Institute of Science and Technology (KIST).

### 2.2. Experimental Paradigm and Bio-Signal Acquisition

**Figure 1:**
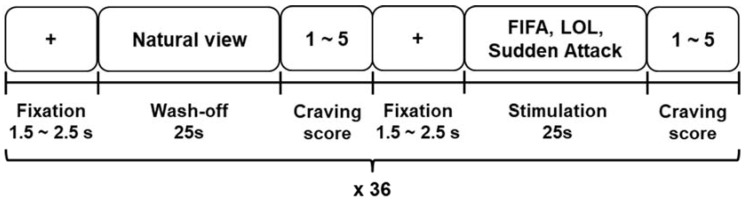
Schematic diagram of experimental paradigm. We alternately presented videos of natural scenery and scenes from three games; FIFA Online 3 (FIFA), League of Legends (LOL), and Sudden Attack. A total of 72 videos were presented to each participant, all of which were different from each other and counterbalanced. In the period, denoted by ‘Craving score’, after the presentation of each video clip, the participants were asked to self-report the strength of their craving for gaming based on the 5-point Likert scale.

We presented 36 short video clips of highly addictive games (referred to as the stimulation trials or stimulation phase) and 36 video clips that were not related to games (referred to as the wash-off trials or wash-off phase) alternately to the participants using a head mounted display (HMD) device (Oculus DK2 HMD; Oculus VR LLC, Menlo Park, CA, USA). Video clips showing dynamic scenes from three addictive games (League of Legends (LOL), Sudden Attack, and FIFA Online 3 (FIFA)) were used, and their appearance frequencies were counterbalanced. Each video was edited to be 25 s long. After watching each video clip, the participants were asked to submit a self-reported craving score according to the 5-point Likert scale based on their subjective feeling of craving for gaming. The self-reported questionnaire was as follows: Please select a number (1–5) best describing your current craving for gaming (1: I do not feel any craving for gaming, 3: I feel craving for gaming, 5: I feel very strong craving for gaming). Figure 1 shows a schematic diagram of the experimental paradigm. Some examples of the video clips used for this experiment can be found at YouTube^TM^ (https://youtu.be/VHYnUWhmOW0).

The PPG, GSR, and EOG signals were recorded using a commercial biosignal recording system (ActiveTwo; BioSemi, Amsterdam, the Netherlands). The PPG and GSR signals were both recorded from the left hand (PPG was acquired from the left index finger; GSR was acquired from the left middle and ring fingers), and the EOG signal was recorded using four flat active electrodes attached around the eyes; these were located at the outer edges of both eyes as well as above and below the right eye. All the signals were recorded at a sampling frequency of 2048 Hz, and the ground and reference electrodes were attached to the left and right mastoids, respectively.

### 2.3. Processing Multiple Biosignals

All the analyses, including signal processing, machine learning, and statistical tests, were performed using MATLAB R2017a (Mathworks, Natick, MA, USA).

#### 2.3.1. Processing of PPG and GSR Signals

For each 25 s epoch, the raw PPG signal was filtered using a 5th order median filter. The respiratory rate (RR) and heart rate (HR) were estimated from the filtered PPG signal using the adaptive infinite impulse response (AIIR) filter-based respiratory rate estimator that we recently developed [19]. To estimate the RR, the signal was down-sampled from 2048 Hz to 128 Hz, and then bandpass filtered using a 4th order Butterworth filter with cut-off frequencies of 0.2 and 0.8 Hz. Two parameters in the RR estimator (*r* and a constant) were set to 0.996 and 3 × 10^−8^, respectively (please refer to [19] for more details). To estimate the HR, the cut-off frequencies of the bandpass filter were set to 2/3 and 4 Hz, and two parameters (*r* and a constant) were set to 0.99 and 6 × 10^−7^, respectively. We estimated the first HR and RR values from the first 10 s signal using a frequency domain analysis, after which we could track the changes in the HR and RR at any time point between 10 and 25 s.

The GSR signal of each epoch was preprocessed using the following procedure: (1) down-sampling of the raw signal from 2048 Hz to 16 Hz; (2) low-pass filtering the signal with a 0.2 Hz cut-off frequency; and (3) linear detrending. Skin conductance responses (SCRs) were extracted by finding the peaks of the preprocessed GSR signal by zero-crossing method, and the mean amplitude of the peaks was calculated [20].

#### 2.3.2. Processing of EOG Signals

Two EOG components (vertical and horizontal components) were acquired from four EOG signals referenced to the right mastoid. The four EOG signals were down-sampled from 2048 Hz to 64 Hz. The vertical EOG component was then obtained by subtracting the signal at the lower edge of the eye from the signal at the upper edge of the eye. The horizontal EOG component was derived by subtracting the left eye signal from the right eye signal. We then applied a median filter with a window size of 7 points to remove noise, and subtracted the median value of each signal to remove the baseline drift [21]. During each video play, we detected eye blinking from the vertical EOG signal using a high-precision eye blink detection algorithm [22]. The detected eye-blink intervals were removed from the vertical EOG and linearly interpolated using the adjacent EOG values. The number of eye blinks was counted and used as one of the candidate features. The horizontal and vertical saccadic eye movements were estimated from two EOG components based on the continuous wavelet transform-saccade detection algorithm [23]. The degree of saccadic movement was evaluated as one of the candidate features by calculating the line integration of the estimated eyeball movement path for each epoch.

### 2.4. Statistical Tests

A parametric or non-parametric statistical test was selected based on the result of the Kolmogorov–Smirnov test, which tests the Gaussianity of a dataset [24]. When the difference between two sets was tested, a paired t-test or Wilcoxon signed rank test was selectively used. When the differences among three or more sets were tested, a one-way analysis of variance (ANOVA) with repeated measures or the Friedman test was selectively used. Bonferroni correction was applied for the multiple comparison correction in the post-hoc analysis.

### 2.5. Classification of Craving States

The SVM was used to classify the high-craving state and low-craving state of each individual. The open software package LIBSVM [25] was used for the classification. In this study, the type of SVM was set at C-SVM, and the cost was set as 10. The radial basis kernel was selected and the gamma in the kernel function was set to {1/the number of features}. We used a total of 14 feature candidates (see Table 1 for the full list of feature candidates; a detailed description of each feature can also be found in Appendix A) and evaluated the classification accuracy using 10-fold cross-validation individually for each participant (64 trials were used for training in each validation). Instead of using specific feature selection methods, we tested all possible combinations of 2–14 features to find the feature set that best fit the training dataset for each fold. We did not use a more efficient feature extraction method because testing all possible feature combinations should be more reliable than using such a method, if the computational cost is not too high. Indeed, testing all possible feature combinations (the number of combinations was 16,369) took about a minute (60.79 s) under a normal personal computer environment (Intel^®^ Core^TM^ i5-6600 Processor @ 3.30 GHz (Intel Corporation, Santa Clara, CA, USA) with a 16 GB RAM). The classification accuracy of each fold of the cross-validation was evaluated using the classifier trained with the selected feature set. The mean classification accuracy of each individual was obtained by averaging the classification accuracies of the 10 cross-validations. We also evaluated the sensitivity and specificity.

## 3. Results

### 3.1. Self-Reported Craving Score

**Figure 2:**
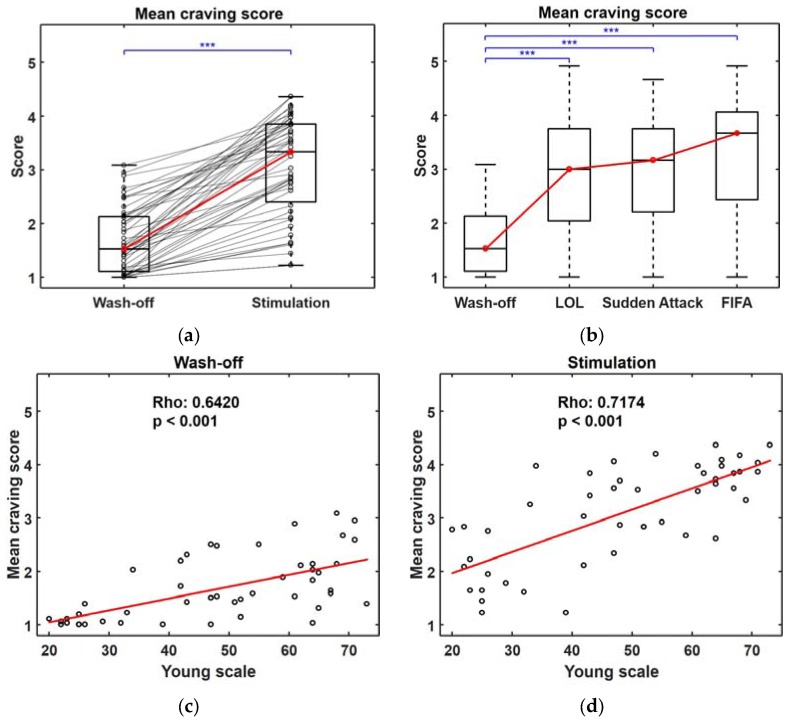
Analysis of the self-reported craving scores of 47 participants. (**a**) Boxplots of mean craving scores after “Wash-off” and “Stimulation” trials. The scores of each participant are indicated using black circles and lines, and the median values of each distribution are indicated using red circles and red lines; (**b**) Boxplots of mean craving scores with respect to different game types. Red circles and lines indicate the median values of each distribution, and *** represents (*p* < 0.001); (**c**,**d**) Relationships between the Young scale and individual participants’ mean craving scores after “Wash-off” and “Stimulation” trials.

We first investigated whether the gameplay videos could effectively arouse the participants’ cravings for gaming by statistically comparing the self-reported craving scores obtained after presenting them with either wash-off videos or gameplay videos. The mean craving score after showing gameplay videos was significantly higher than that after playing wash-off videos, as shown in Figure S1a of the supplementary materials (*p* < 0.001). However, the mean craving scores were not significantly different among the different games, as shown in Figure S1b of the supplementary materials. These results suggest that the gameplay videos used for stimulation were effective at arousing cravings for gaming. In order to compare the biosignals recorded in the craving and non-craving states, we excluded 10 participants (17.54% of 57 participants) whose self-reported craving scores for the two different types of trials (i.e., the stimulation and wash-off trials) were not significantly different or whose mean craving score after stimulation was lower than that after watching the wash-off videos (see Supplementary Table S2). Figure 2a,b shows boxplots of the mean craving scores after excluding these 10 participants, where there is still no significant difference among the three game types, suggesting that the contents of the games did not influence the degree of aroused craving. We also evaluated the correlation between the participants’ mean craving scores and Young’s internet addiction test scores (Young scale) to determine whether the self-reported craving scores had a close association with IGD. Figure 2c,d shows scatter plots of the individual craving scores acquired after showing the wash-off videos and gameplay videos, respectively. In both cases, statistically significant correlations were found between the Young scale and the mean craving score, implying that a stronger craving is aroused when the severity of IGD is higher. Even in the baseline (wash-off) condition, individuals with severe IGD showed high levels of craving for gaming. The Spearman correlation coefficient was slightly higher in the craving score after stimulation (Rho: 0.72, *p* < 0.001) compared to that of the craving score after the wash off (Rho: 0.64, *p* < 0.001).

### 3.2. Changes in Multiple Biosignals When Craving for Gaming Was Aroused

**Figure 3:**
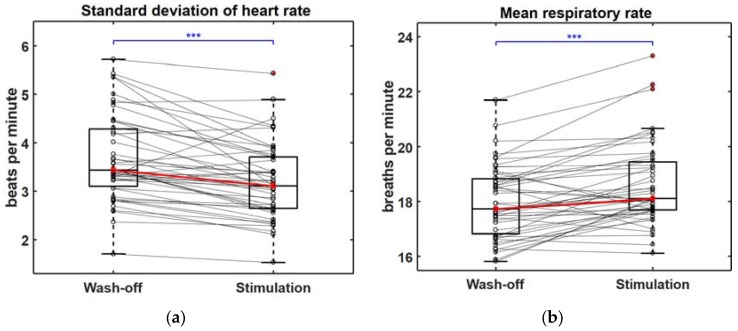
Differences in basic features of autonomic nervous system responses recorded during “Wash-off” and “Stimulation” trials, where (**a**,**b**) show changes in standard deviation of heart rate and mean respiratory rate, respectively, and *** indicates (*p* < 0.001).

**Figure 4:**
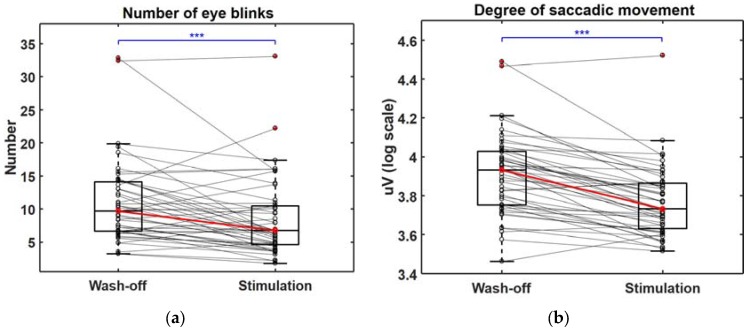
Differences in basic features acquired from electrooculogram during “Wash-off” and “Stimulation” trials, where (**a**,**b**) show changes in number of eye blinks and degree of saccadic movement, respectively; and *** indicates (*p* < 0.001).

Changes in the biosignals recorded during the gameplay video screening were observed. During the stimulation trials, the standard deviation of the heart rate was significantly decreased (*p* < 0.001; Figure 3a) and the mean respiratory rate was significantly increased (*p* < 0.001; Figure 3b). We also found significant decreases in the number of eye blinks (*p* < 0.001; Figure 4a) and the saccadic movement distance (*p* < 0.001; Figure 4b) estimated from the EOG signals. The amplitude of the SCR was decreased during the stimulation trials, but did not show statistical significance (*p* = 0.0625; Figure S2a of the supplementary materials). However, a secondary feature derived from the SCR, namely, the mean amplitude of the normalized SCR, showed a significant decrease during the stimulation trial (*p* < 0.001; Figure S2b of the supplementary materials). We also investigated the relationship between each feature and the Young scale, and found significant correlations between four EOG-derived features (DSM, DVSM, mDHV, and CHV) and the Young scale (see Supplementary Figure S3a--d for the scatter distributions and the correlation coefficient values). In addition, we compared the mean values of each feature among the three games, but no significant differences were found. The distributions of all the other features used for the classification of the craving states (9 out of 14 features) can be found in Figure S4 of the supplementary materials, with the other five features depicted in Figure 3a,b, Figure 4a,b and Figure S2b. All of the features, except the mean heart rate (mHR) and standard deviation of the respiratory rate (stdRR), showed statistically significant differences between the two conditions (*p* < 0.001). Note that the statistical significance (*p* < 0.05) of all the features were still preserved even after applying Bonferroni correction (*N* = 14) because they all showed a low probability of *p* < 0.001 before applying the Bonferroni correction. Also, the difference in stimulation and wash-off trials for 3 out of 9 features in Figure S4 showed significant correlation with the Young scale in Figure S3b–d of the supplementary materials.

### 3.3. Classification of Craving State Using Machine Learning

**Figure 5:**
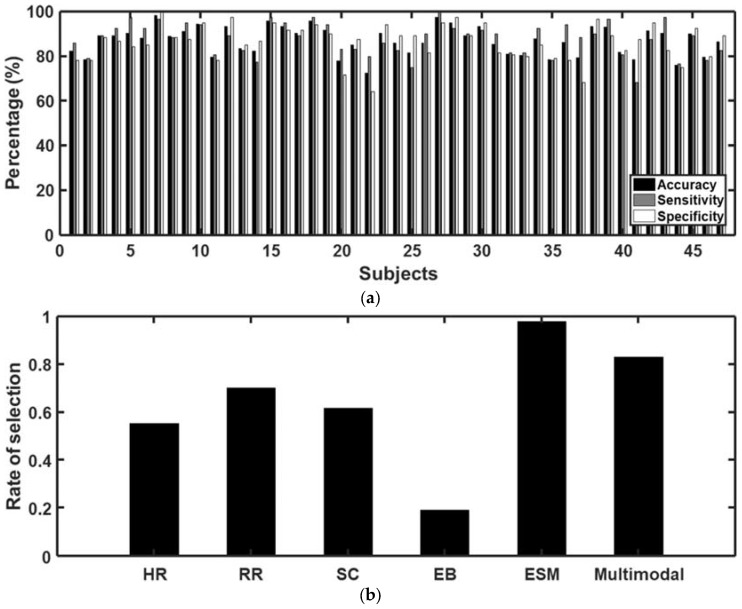
Results of classification of craving states. (**a**) Performance of the binary classification of craving states validated through 10-fold cross-validation. Black, grey, and white bar graphs indicate the average accuracy, sensitivity, and specificity, respectively. The average accuracy was 87.04%, when the sensitivity and specificity were 87.71% and 86.37%, respectively; (**b**) The rate of selection of each kind of feature in the classification. HR, RR, SC, EB, ESM, and Multimodal represent the heart rate, respiratory rate, skin conductance, eye blink, eye saccadic movement, and multimodal covariance features, respectively. The detailed results are presented in Table S1 of the supplementary materials.

Although two (mHR and stdRR) of the fourteen feature candidates did not exhibit statistical differences between the two different types of trials, all the feature candidates were used for the classification because they still had the potential to provide important information for the classification. Figure 5a and Supplementary Table S1 show the accuracy of classifying the craving states using the multimodal biosignals acquired from each of the 47 participants. The average classification accuracy was 87.04%, and the average sensitivity and specificity were 87.71% and 86.37%, respectively. We also investigated the correlation between the classification performance (accuracy, sensitivity and specificity) and the Young scale, but the analysis results did not exhibit any significance (Accuracy: Rho = 0.1830, *p* = 0.2183; Sensitivity: Rho = 0.1259, *p* = 0.3991; Specificity: Rho = 0.1898, *p* = 0.2013), suggesting that the classification performance is not affected by the severity of IGD symptoms. Figure 5b shows the selection rate for each feature type after the feature selection (see also Table S3 of the supplementary materials for more detailed results), from which it can be observed that features estimated from the eye saccadic movement were most frequently selected. Interestingly, two features that did not show meaningful differences in the group statistical analysis (i.e., mHR and stdRR) were selected more frequently than some other features that showed statistical significance in the group analysis.

## 4. Discussion and Conclusions

We presented adolescents who had IGD with two types of short video clips, online gameplay and natural scenery, to arouse and diminish the craving for gaming, respectively. Although most of the paradigms introduced in previous craving studies use still picture images related to addictive objects and neutral scenery images [7,26,27,28], a previous study demonstrated that videos related to addictive objects can also effectively induce a craving [9]. In this study, the increase in the self-reported craving score after watching gameplay videos indicated that the gameplay videos used for the experiments were effective enough to make the participants feel a craving for gaming. Interestingly, this craving for gaming was aroused by the gameplay videos in most participants regardless of the severity of their IGD; however, individuals with higher Young scales generally reported higher craving scores, suggesting that individuals with severe IGD are apt to feel a stronger craving for gaming than those with mild IGD. These results are in line with the results of a previous addiction study performed with drug-dependent individuals [4]. 

Some representative features were evaluated using the multimodal biosignals recorded while presenting either gameplay videos or natural scenery videos. In general, the heart rate, respiratory rate, and skin conductance are regarded as indicators reflecting the activation of the autonomic nervous system. When the sympathetic nervous system becomes activated, the standard deviation of the heart rate is decreased, and the respiratory rate and skin conductance are increased [12,29]. In the craving condition, a decrease in the standard deviation of the heart rate and increase in the respiratory rate were observed, as in previous studies, indicating the activation of the sympathetic nervous system. However, the amplitude of the skin conductance decreased, although there was no statistical significance. Lu et al. explained that this opposite directional variation of skin conductance was a result of an aversive feeling associated with withdrawal [12]. An electroencephalography (EEG) study also reported negative affect experienced by heavy smokers when craving was aroused by smoking-related cues [30]. Therefore, the decrease in skin conductance might be a reflection of the negative affect related to the withdrawal symptoms experienced by the participants with IGD.

Although changes in heart rate, respiratory rate, and skin conductance during the craving state have been reported in previous addiction studies, no previous study has attempted to use EOG signals as potential markers of the craving state. In this study, the number of eye blinks was significantly decreased during the gameplay video screening, which might reflect the increased attention of the participants toward the addictive objects; the number of eye blinks has been known to be closely associated with attention levels [31,32,33]. It was also found that the distance of the saccadic movements was significantly decreased during the stimulation trials compared to the wash-off trials. Before the experiments, it was expected that the distance of the saccadic movements might be increased because the gameplay videos contained more dynamic scenes than the wash-off videos; however, a totally opposite result was obtained in our experiments. Mogg et al. demonstrated that smokers stared at smoking-related pictures longer than control pictures by measuring the direction and duration of their gaze. They concluded that a craving for smoking induced a biased attentional orientation to smoking cues [34]. Therefore, decreases in the number of eye blinks and the distance of the saccadic movements might be associated with the increased attention due to the increased cravings for gaming. Therefore, these features are expected to be potentially useful for indirectly measuring an individual’s degree of craving for gaming, especially when audiovisual stimuli are presented. It is noteworthy that the distance of the saccadic movements was the most frequently selected feature in the machine learning-based classification of the craving state. This result is in line with the results of correlation analyses, in which only saccadic-movement-related features showed significant correlations with the Young scale. These results suggest that EOG-based features might better reflect the characteristics of IGD than other features based on the autonomic nervous system responses.

**Figure 6:**
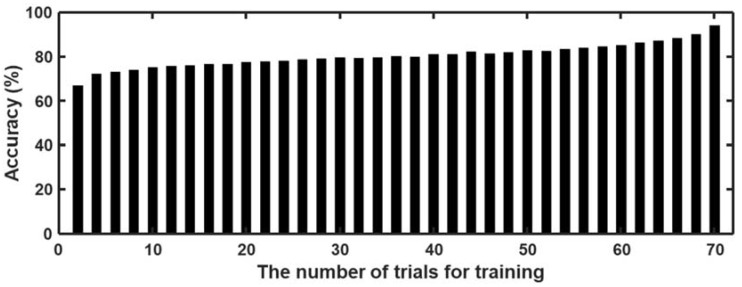
Changes in average accuracy with respect to number of trials used for training.

Many addiction-related studies have investigated the biosignal variations elicited by craving-inducing stimuli or investigated the difference between the biosignal responses of control and addiction groups [12,34,35]. However, to the best of our knowledge, no study has estimated or classified an individual’s current craving state. In the present study, we attempted to classify whether or not an individual was craving using 14 biosignal-derived features. Because the mean craving score of the stimulation trials was significantly higher than that of the wash-off trials, we assumed that the stimulation trials produced a craving state, and the wash-off trials produced a non-craving state. As a result of the SVM-based classification, we could distinguish the craving state from the non-craving state with a fairly high classification accuracy of 87%. Although the average accuracy of the binary classification evaluated using the 10-fold cross-validation was high, the number of training trials needs to be minimized to facilitate the practical use of this craving state detection method. Figure 6 shows the changes in accuracy with respect to the number of training trials. Each accuracy value represents the mean accuracy averaged across 47 participants, where the accuracy of each participant was evaluated by repeating the classification procedure 10 times with randomly chosen training trials. Based on the analysis results, it could be seen that a fairly high classification accuracy (as high as 75%) could still be achieved even when only five training trials were used to build the classifier.

In this study, we observed the changes in diverse autonomic nervous system responses, as well as the autonomic responses of eyeball/eyelid movements, when adolescents with IGD were exposed to game-related audiovisual stimuli. Based on observations of the changes in the biosignals acquired with PPG, GSR, and EOG measurements, whether or not a participant was in a craving state was classified with a fairly high classification accuracy. However, this study did not take EEG into account, which has been widely used in cue-elicited craving studies [30,36]. Therefore, a combination of EEG-driven features and biosignal-driven features may have the potential to increase the overall classification accuracy, which we would like to investigate in our future studies. In addition, in the near future, we are planning to apply the developed craving state detection method to neurofeedback therapy for IGD, which requires the precise real-time detection of the craving state. A critical limitation of our study is that about a sixth of individuals with IGD might not be eligible to use the real-time neurofeedback system. Indeed, we excluded 10 (out of 57) participants in our analyses because our stimulation videos were not very effective in arousing craving for gaming for these participants and thus, the biosignals recorded from them were thought to be inadequate to be used for making reliable classifiers. To address this issue, more effective ways to arouse craving for gaming to most individuals with IGD need to be developed in future studies. Another limitation of the current study is the relatively short inter-stimulus interval (ITI), the average of which was 6.27 s. Because skin conductance response may sometimes have longer half-recovery time (generally from 2 s to 10 s) than this [37], slow recovery of the skin conductance response might have a potential impact on the analysis results in some participants. Although such influence might be limited considering that the duration (25 s) of each trial was much longer than the difference between the ITI and the half-recovery time, this issue needs to be more concretely addressed using new experimental data in the future study. On the other hand, there are a variety of features quantifying heart rate variability (HRV) that can potentially provide a more specific evaluation of the sympathovagal balance than the basic features used in this study. Because many HRV features are reliable when calculated over a 5-min heartbeat signal [38], these long-term features were not suitable for our experiments with 25-s trials. In the case of the HF component (0.15 to 0.4 Hz) of HRV, recording for approximately 1 min is needed to precisely assess this feature [39]. A longer recording time would be necessary in future studies to investigate whether various HRV features can effectively detect craving for gaming in individuals with IGD.

## Figures and Tables

**Table 1:** List of features used for binary classification. Each column presents the name (unit) of each feature and a brief description of it. More detailed descriptions of the features can be found in Appendix A.

Feature (Unit)	Description	Feature (Unit)	Description
stdHR (beats per minute)	Standard deviation of heart rate	DHSM (µV)	Degree of horizontal saccadic movement
mHR (beats per minute)	Mean heart rate	DVSM (μV)	Degree of vertical saccadic movement
stdRR (breaths per minute)	Standard deviation of respiratory rate	mDHV (μV)	Mean of DHSM and DVSM
mRR (breaths per minute)	Mean respiratory rate	DSM (μV)	Degree of saccadic movement
mNSC (no unit)	Mean amplitude of normalized skin conductance response	CHV (μV^2^)	Covariance of horizontal EOG and vertical EOG
minNSC (no unit)	Minimum amplitude of normalized skin conductance response	CHP (μV)	Covariance of horizontal EOG and PPG
NE (no unit)	The number of eye blinks	CVP (μV)	Covariance of vertical EOG and PPG

## Supplementary Materials

The following are available online at http://www.mdpi.com/1424-8220/18/1/0102/s1: Figure S1: Boxplots of mean craving score of 57 subjects before excluding 10 subjects who did not show typical changes in craving score, Figure S2: Differences in features derived from galvanic skin response (GSR) recorded during “Wash-off” and “Stimulation” trials, Figure S3: Relationship between four EOG-based features and the Young scale, Figure S4: Differences in nine features (out of 14 features) evaluated for “Wash-off” and “Stimulation” trials, Table S1: Young scales, craving scores, and classification performance of 47 subjects, Table S2: Young scales and craving scores of 10 subjects excluded in the analyses, Table S3: Rates of selection of 14 features of 6 types in binary classification.

## Appendix A. Calculation of Features Listed in Table 1

To calculate the mean amplitude of the normalized skin conductance response (mNSC) and the minimum amplitude of the normalized skin conductance response (minNSC), we normalized the preprocessed galvanic skin response signals using *z*-score transformation and found the peaks from the normalized signals by zero-crossing method. mNSC is the mean amplitude of the peaks, and minNSC is the minimum amplitude of the peaks.

The number of eye blinks (NE) and four similar kinds of degrees of saccadic movements were calculated from an electrooculogram (EOG). The methods used to detect eye blinks and saccadic movements were described in Section 2.3.2. NE was calculated by counting the number of detected eye blinks. The degree of horizontal saccadic movement (DHSM) and degree of vertical saccadic movement (DVSM) were computed by summing up (numerically integrating) the absolute changes in the saccadic movements estimated from horizontal and vertical EOGs, respectively. mDHV indicates the mean of DHSM and DVSM. The degree of saccadic movement (DSM) was calculated by the numerical line integration of the estimated eyeball movement path estimated from both horizontal and vertical EOGs.

CHV, CHP, and CVP are the values of covariance among the horizontal EOG, vertical EOG, and PPG. The three signals were first preprocessed as described in Section 2, without the down-sampling process (sampling frequency: 2048 Hz). The covariance was calculated using the following equation:

(A1) $$\mathrm{COV}\left(X,Y\right)=\;\frac{1}{N-1}\sum_{i=1}^{N}{\left(X_{i}-\;\bar{X}\right)}^{*}(Y_{i}-\;\bar{Y}),$$

where $\bar{X}$ and $\bar{Y}$ are the mean values of signal X and signal Y, respectively; N is the length of the signal; and * indicates the complex conjugate.
