# Supplementary material for: Detection of Craving for Gaming in Adolescents with Internet Gaming Disorder Using Multimodal Biosignals
Source: Sensors (Basel). 2018 Jan 1;18(1):102. doi: 10.3390/s18010102 (PMC5795403; doi:10.3390/s18010102)
Supplement: Supplementary file 1 [file sensors-18-00102-s001.pdf]

Detection of Craving for Gaming in Adolescents with Internet Gaming Disorder Using Multimodal Biosignals

Hodam Kim, Jihyeon Ha, Won-Du Chang, Wanjoo Park, Laehyun Kim, and Chang-Hwan Im

Table S1. Young scales, craving scores, and classification performance of 47 subjects. The scores in "Wash-off" and "Stimulation" columns indicate the mean values of each type of trials.

Subject	Young Scale	Craving Score		Classification Performance (%)		
		Wash-Off	Stimulation	Accuracy	Sensitivity	Specificity
1	48	2.47	3.69	82.44	85.83	78.33
2	52	1.47	2.83	78.51	79.17	78.33
3	43	1.42	3.42	89.29	89.17	88.33
4	52	1.14	2.83	89.29	92.5	86.67
5	64	1.03	2.61	90.36	97.5	84.17
6	42	1.72	2.11	88.04	92.5	85
7	65	1.31	3.97	98.33	96.67	100
8	64	1.83	4.36	88.93	88.33	88.33
9	42	2.19	3.03	91.13	95	87.5
10	47	1	4.06	94.46	94.17	95
11	33	1.22	3.25	79.7	80.83	78.33
12	51	1.42	3.53	93.39	89.17	97.5
13	54	1.58	4.19	83.45	82.5	85
14	25	1.19	1.44	82.26	77.5	86.67
15	67	1.64	3.83	95.89	97.5	95
16	68	2.14	4.17	93.33	95	91.67
17	59	1.89	2.67	90.42	89.17	91.67
18	34	2.03	3.97	95.89	97.5	94.17
19	48	1.53	2.86	91.73	94.17	90
20	67	1.58	3.56	77.92	83.33	71.67
21	69	2.67	3.33	85.12	83.33	87.5
22	25	1	1.64	72.5	80	64.17
23	32	1.03	1.61	90.24	85.83	94.17
24	26	1.39	2.75	85.83	82.5	89.17
26	23	1.03	2.22	81.43	75	89.17
27	55	2.5	2.92	85.89	90	81.67
28	29	1.06	1.78	97.5	100	95

29	43	2.31	3.83	95	92.5	97.5
31	47	2.5	3.56	89.29	90	89.17
32	65	1.97	4.08	93.33	91.67	95
33	39	1	1.22	85.48	90	81.67
34	71	2.94	4.03	80.95	81.67	80.83
37	23	1.11	1.64	80.48	81.67	80
39	22	1	2.83	87.86	92.5	85
40	64	2.14	3.72	78.63	78.33	79.17
41	25	1	1.22	86.13	94.17	78.33
42	73	1.39	4.36	79.4	88.33	68.33
46	62	2.11	3.83	93.21	90	96.67
47	64	2.03	3.64	92.98	96.67	89.17
48	47	1.5	2.33	81.9	80.83	82.5
49	26	1	1.94	78.57	68.33	87.5
50	71	2.58	3.86	91.55	87.5	95
53	22	1.06	2.08	90.48	97.5	82.5
54	20	1.11	2.78	76.01	76.67	75
55	68	3.08	3.86	90.12	89.17	92.5
56	61	1.53	3.97	79.64	78.33	80
57	61	2.89	3.5	86.37	82.5	89.17

Table S2. Young scales and craving scores of 10 subjects excluded in the analyses. The scores in "Wash-off" and "Stimulation" columns indicate the mean values of each type of trials. The significance represents the result (p-value) of paired t-test comparing the self-reported craving scores obtained after wash-off and stimulation trials for each subject.

**Subject**	**Young Scale**	**Craving Score**		
		**Wash-Off**	**Stimulation**	**Significance**
25	72	2.81	3.06	0.095
30	76	3.53	3.22	0.145
35	26	1.03	1.08	0.314
36	76	4.75	1	0
38	28	1	1.11	0.082
43	75	4.14	4.03	0.545
44	21	1.94	2.19	0.272
45	71	3.33	3.75	0.109
51	23	1	1.03	0.331
52	23	1	1	X

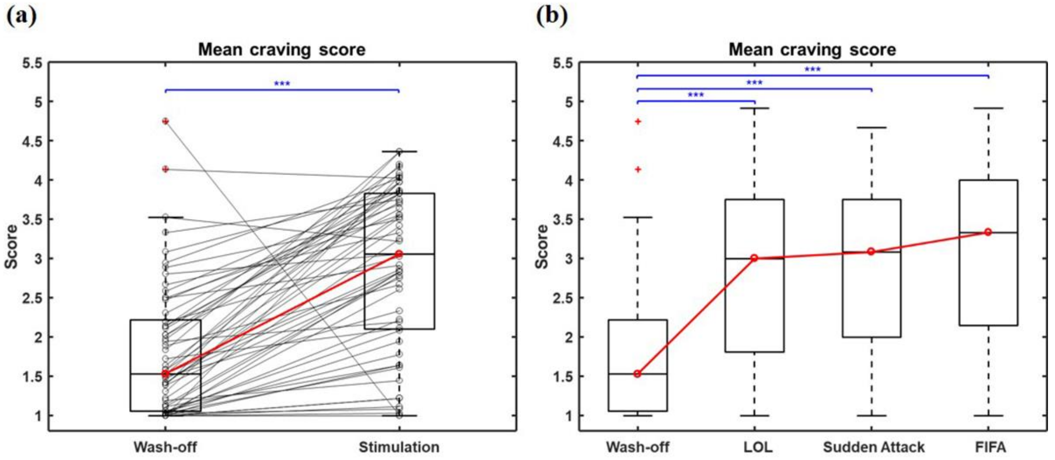

Figure S1. Boxplots of mean craving score of 57 subjects before excluding 10 subjects who did not show typical changes in craving score. (**a**) Boxplots of mean craving scores after "Wash-off" and "Stimulation" trials. Scores of each participant are indicated using black circles and lines and the median values of the distributions are indicated using red circles and red lines. (**b**) Boxplots of mean craving scores with respect to different game types. Red circles and lines indicate the median values of the distributions, where *** represents ($p < 0.001$).

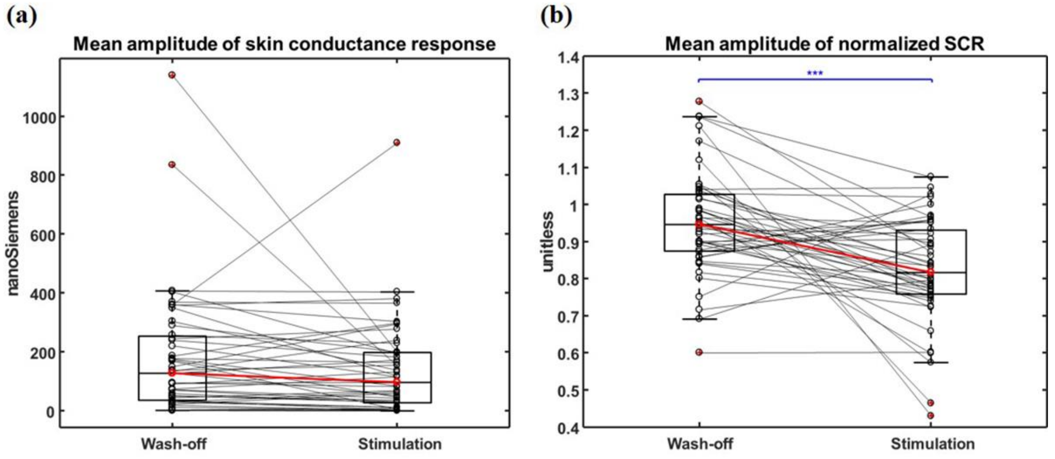

Figure S2. Differences in features derived from galvanic skin response (GSR) recorded during "Wash-off" and "Stimulation" trials, where (**a**) and (**b**) show changes in mean amplitude of skin conductance response (SCR) and mean amplitude of normalized SCR (mNSC), respectively; and *** indicates ($p < 0.001$).

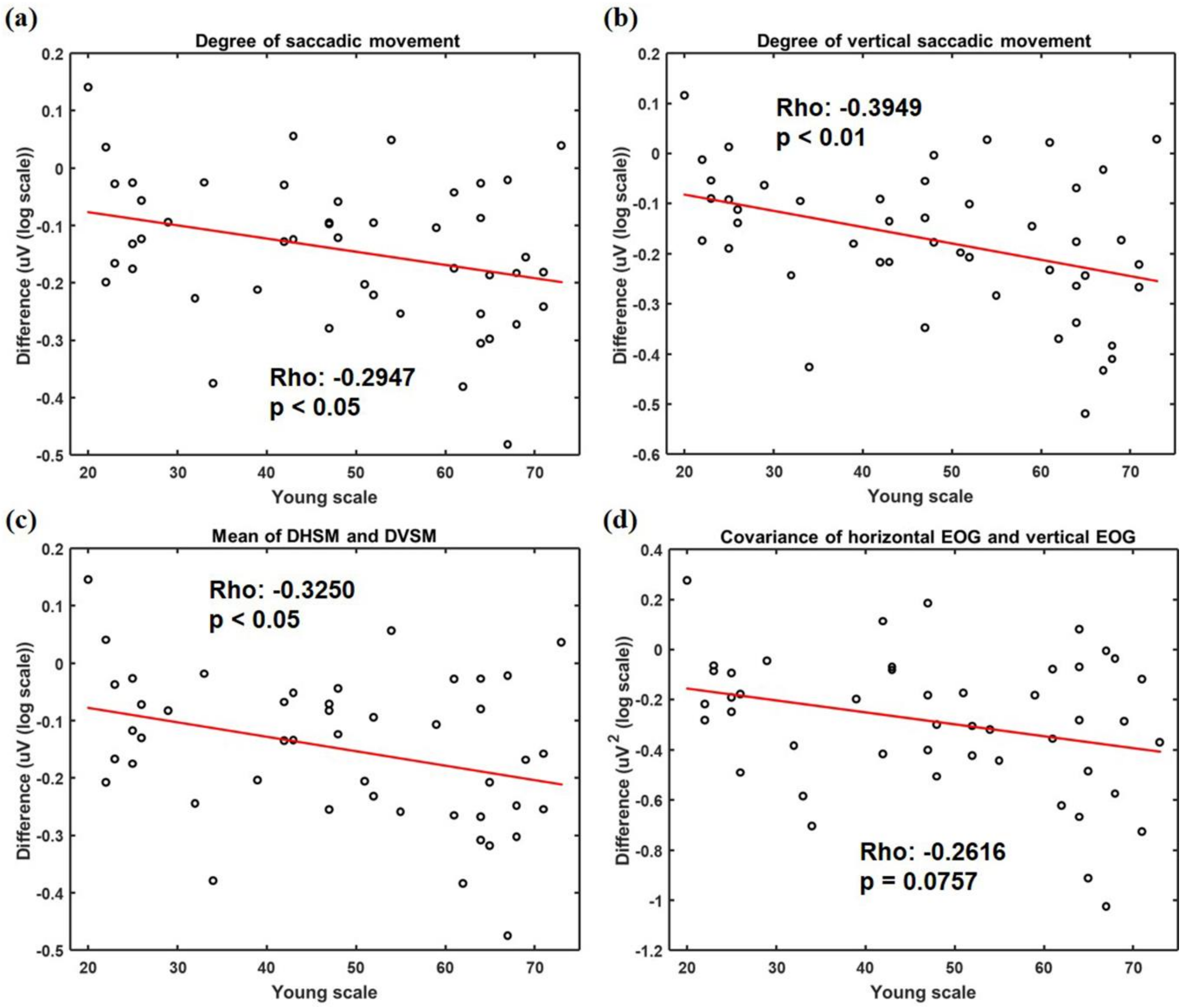

Figure S3. Relationship between four EOG-based features and the Young scale. "Difference" in y-axis indicates the difference between mean feature values of stimulation and wash-off trials: (**a**) DSM, (**b**) DVSM, (**c**) mDHV, (**d**) CHV.

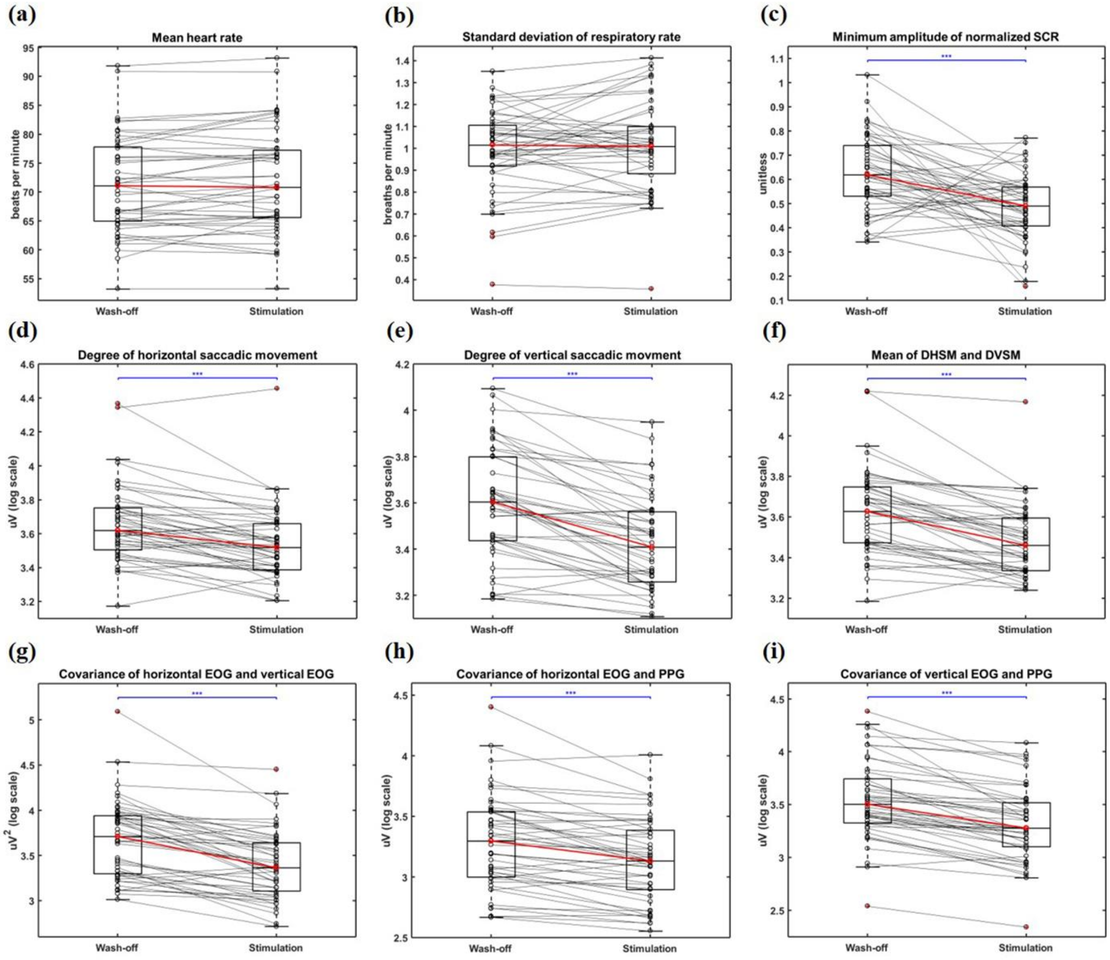

Figure S4. Difference in nine features (out of 14 features, with boxplots of other five features presented in Figures 3a,b, 4a,b, and S2b) evaluated for "Wash-off" and "Stimulation" trials: (**a**) mHR, (**b**) stdRR, (**c**) minNSC, (**d**) DHSM, (**e**) DVSM, (**f**) mDHV, (**g**) CHV, (**h**) CHP, and (**i**) CVP. Black circles indicate the mean values of each participant and the red circles indicate the median of the individual mean values, with *** representing ($p < 0.001$). The results in (**a**) and (**b**) do not show any statistically significant difference.

Table S3. Rates of selection of 14 features of 6 types in binary classification. The rate of a type increases when any one of its features is selected as a component of a combination with the highest accuracy for a subject and the rate of a feature increases when the feature is selected as a component of a combination with the highest accuracy. The rate is one when a type or feature is selected for every subject (HR: heart rate, RR: respiratory rate, SCR: skin conductance response, NE: the number of eyeblinks, ESM: eye saccadic movement).

Type	Rate	Feature	Rate	Type	Rate	Feature	Rate
HR	0.55	stdHR	0.38	ESM	0.98	DHSM	0.51
		mHR	0.32			DVSM	0.49
RR	0.70	stdRR	0.47			mDHV	0.51
		mRR	0.36			DSM	0.49
SCR	0.62	mNSC	0.47	Multi-modal	0.83	CHV	0.53
		minNSC	0.40			CHP	0.28
NE	0.19	NE	0.19			CVP	0.49
